# Reduction of response time by data placement reflecting co-occurrence structures in structured overlay networks

**DOI:** 10.1371/journal.pone.0205757

**Published:** 2018-10-12

**Authors:** Yusuke Koizumi, Kohei Watabe, Kenji Nakagawa

**Affiliations:** Graduate School of Engineering, Nagaoka University of Technology, Nagaoka, Niigata 940-2188, Japan; Max Stern Yezreel Valley College, ISRAEL

## Abstract

We propose a method to accelerate a response of structured overlay networks by reducing the number of hops required to answer multi-queries. In the proposed method, by copying data items to the redundant storage spaces in other storages, a good data placement reflecting co-occurrence structures in the structured overlay network is achieved. We formulate the optimization problem of the data placement in the limited redundant space of the storages as an integer programming. A greedy approach to solve the optimization problem is also proposed. Through several simulations, it is confirmed that the proposed method can reduce the average number of hops required to answer multi-queries by about 30% at the maximum in our simulation settings. The reduction rate of the average number of hops depends on the level of co-occurrence. Further, the reduction of the computation time to solve the optimization problem with the greedy approach is evaluated. We also confirm that the proposed method does not affect load balancing of structured overlay networks.

## 1 Introduction

In recent years, the utilization of big data attracts attention, and various services that handle huge data collected from various devices such as sensors are expected. There are several technologies that support these big data services, such as inexpensive sensors. Even among them, storage systems for storing and managing the huge amount of collected data are one of the key technologies.

It is expected that storage systems based on structured overlay networks can solve a scalability problem in big data management [1–7]. In a storage system based on a structured overlay network, an overlay network is constituted by a large number of storages on an underlay network (typically, on the Internet), and data items are distributed on these storages. There is no central server that indexes data items stored on each storage since systems are decentralized. Therefore, a user cannot find a data item by querying to a central server. When a storage receives a query from a user, the system searches a storage that stores the data item in the query by repeatedly forwarding the query to a neighbor node.

One of the most important performance measure in storage systems based on structured overlay networks is a response time to a query. However, in a multi-query requiring a large number of data items at the same time, a response time to a multi-query can be delayed. In structured overlay networks, since required data items from a multi-query are distributed in multiple storages, it is necessary to search a large number of storages.

Reflecting co-occurrence structures of all stored data items on a structured overlay network is important for reducing response time to multi-queries. Generally, stored data items include co-occurrence structures. A specific combination of data items may always be required together. These data items should be placed at the same storage to reduce response time since these data items can be found by one search. Conversely, these data items may tend to not be required with other data items, simultaneously. Such data items do not necessary to place at the same storage. It is expected that a response time to a query can be reduced by reflecting co-occurrence structures on data placement of structured overlay networks.

Some protocols in which a part of the co-occurrence structures reflect on placement of data items has been proposed in the literature on structured overlay networks. In protocols that support range-query such as Chord# [7] and Mercury [1], we can consider that a co-occurrence structure is expressed by a one-dimensional ID space. In range-query, required data items are specified by a range of data IDs, and data items with adjacent IDs are required together. In order to support range-queries, data items with adjacent IDs are placed at the same storage in most cases. Additionally, the extension of ID space to a multi-dimensional space has also been studied [8, 9].

However, these conventional approaches in which co-occurrence structures are expressed by ID space do not fully reflect the structures onto the placement of data items. In some cases, co-occurrence structures may be complex, and they cannot be mapped into simple ID space such as a one or multi-dimensional space. Moreover, they may change dynamically, and the change is unexpected. In structured overlay networks, an ID space of data is designed before an application is released. It is hard to change the ID space after the release of the application. As the application growth and spread widely, the co-occurrence structure may be changed depending on the behavior of users. The conventional approaches cannot follow the dynamical change of the co-occurrence structures since the user behavior is difficult to predict.

In this research, we propose a novel method to reduce response time of multi-queries by reflecting co-occurrence structures of all stored data items onto the data placement in structured overlay networks. The proposed method extends a conventional routing method (e.g., Chord [4]) on structured overlay networks. Data items are copied to other storages in consideration of co-occurrence structures which are not reflected in the ID space. A conventional routing method can work consistently since source items of copied data items are left at the original storage. We formulate an optimization problem for data placement as an integer programming. We also propose a greedy algorithm that solves it, thereby minimizing the response time of multi-queries in space limited storages. Each storage performs in decentralized manner only with local information, though the optimum data placement is achieved. The proposed method works with most of conventional routing methods, including Chord [4], Chord# [7], and Mercury [1] without conflict.

The remainder of this paper is organized as follows. Section 2 introduces conventional routing methods that are base of our proposed method. We explain the formulation of the problem and the greedy algorithm that optimizes the placement of data items in Section 3. In Section 4, we evaluate the proposed method by comparing with a placement based on a simple Least Recently Used (LRU) approach. We summarize related works in Section 5. Section 6 concludes the paper and presents future research directions.

## 2 Structured overlay networks

**Fig 1:**
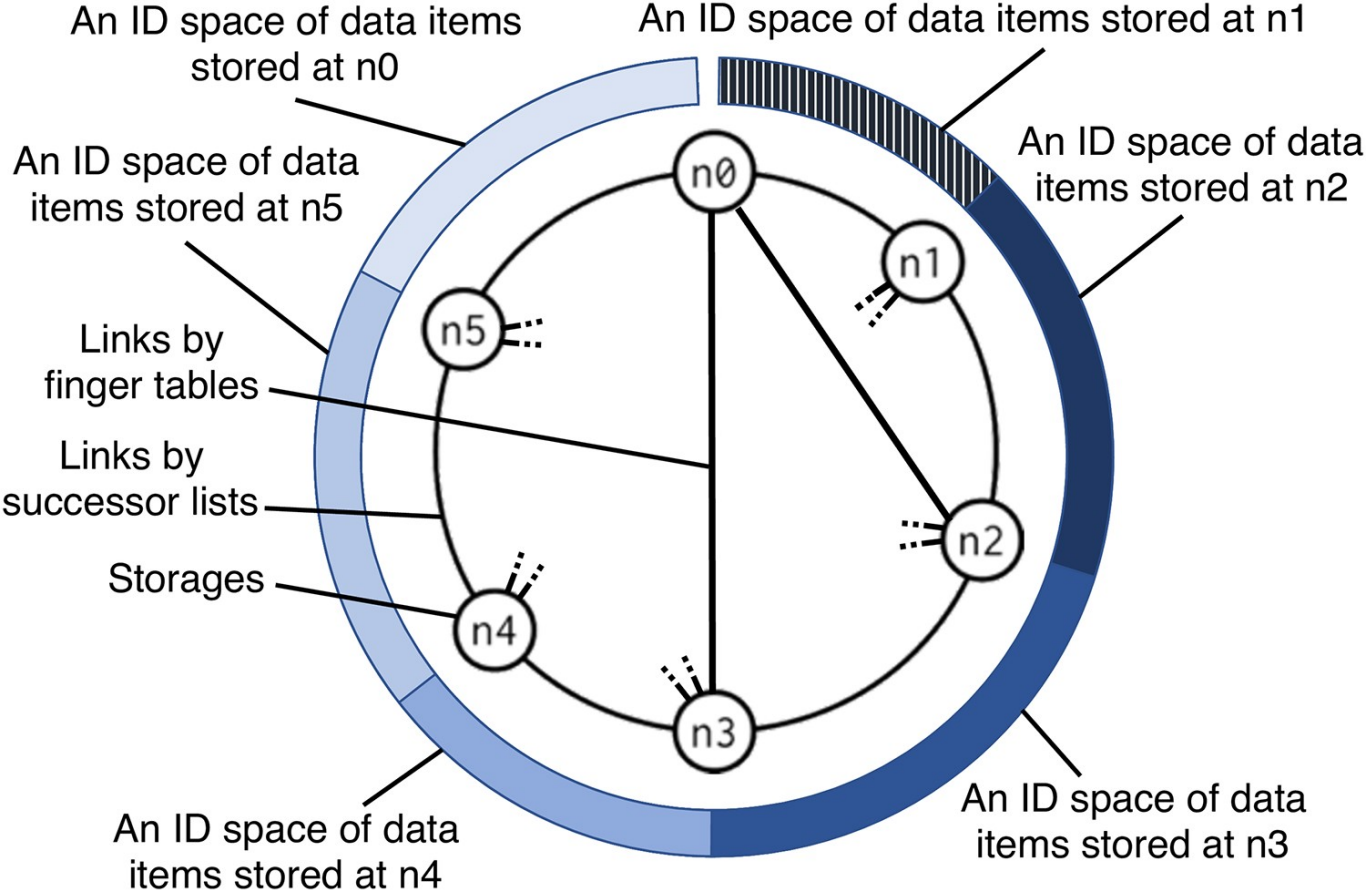
A topology of a overlay network and ID space in Chord or Chord#.

In structured overlay networks, an overlay network that has a specific topology (e.g., a ring topology) is constructed on an underlay network, by connecting storages each other with virtual links. The data items are distributed between many storages and each storage performs in decentralized manner, thereby being able to handle huge amount of data items. In Chord that is a representative protocol of structured overlay networks, the storages form a ring network by maintaining successor lists (see Fig 1). Additionally, by maintaining finger tables, storages that are far away in the ring network are connected by virtual links in order to reduce the number of hops to a data item. The ID space of data is a one-dimensional space in Chord, and a storage in the ring network stores data items in a part of the ID space. Since data ID is assigned using a hash function, a load of each storage is balanced.

Some protocols support range-queries in which required data items are specified by a range of data IDs [5, 7, 9–11]. Chord# extends Chord for range-queries by replacing the hash function to a key-order preserving function. It constructs a ring network and a one-dimensional ID space as with Chord. Since load balancing effect by hashing is lost in Chord#, it adopts load balancing technique proposed by D. Karger *et al*. [12]. When range-queries are effective, data items with adjacent IDs tend to be required together. In other words, data items with adjacent IDs have high co-occurrence. In order to support range-queries, data items with adjacent IDs are placed at the same storage (or the adjacent storages) in Chord#. It means that the co-occurrence structure is expressed as a one-dimensional ID space, thereby reflecting the co-occurrence structure to the placement of data items.

The number of hops required to answer a query is one of the most important performance metrics in structured overlay networks. Each storage performs in decentralized manner, and there is no central server that indexes data items stored on each storage. Required data items are searched by repeatedly forwarding a query to a neighbor node.

## 3 A data copy method reflecting co-occurrence structures

In this section, we will explain the proposed method to reduce the response time to multi-queries by reflecting co-occurrence structures of all stored data items onto the data placement in structured overlay networks. In the proposed method, we place copies of data items at storages that do not have original data items, thereby increasing the probability of getting data items in a multi-query at the same storages. We formulate the optimization problem of the placement of the copied data items in the limited space of storages as an integer programming. Moreover, we propose a greedy algorithm that solves it.

### 3.1 Co-occurrence structures of data items

**Fig 2:**
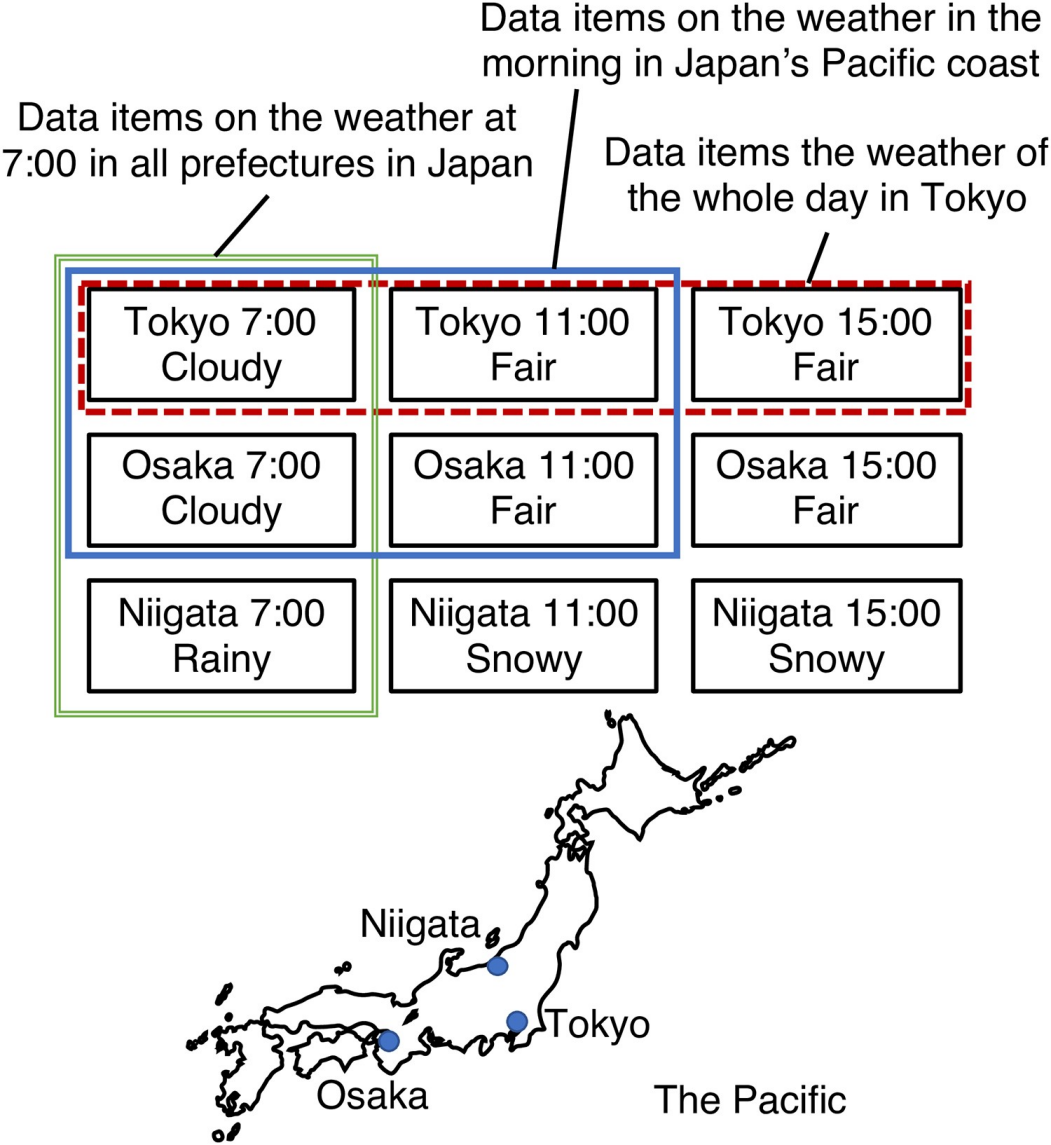
An example of co-occurrence of data items.

There are combinations of data items that tend to be required at the same time in data items stored on structured overlay networks, and we call the combinations as co-occurrence structures. These co-occurrence structures can affect performance of structured overlay networks. We show an example of co-occurrence structures that is shown in Fig 2. In Fig 2, we consider the case where weather information at 7:00, 11:00, and 14:00 in Tokyo, Osaka, and Niigata are stored on a structured overlay network. Let us focus on combinations of data items surrounded by 3 rectangles: 1) The data items in the red dotted line rectangle can be regarded as data items on the weather of the whole day in Tokyo, 2) The data items in the green double line rectangle can be regarded as data items on the weather at 7:00 in all prefectures in Japan, 3) The data items in the blue solid line rectangle can be regarded as data items on the weather in the morning in Japan’s Pacific coast. In this example, we assume that these combinations of data items tend to be required at the same time.

If we placed the data items in these combinations at the same storage, the number of hops required to answer a query can be reduced. When we can expect these tendencies as a prior knowledge (the tendency of case 1) and 2) are easily expected), we can utilize the conventional methods that support range-queries. However, it is difficult to expect all the co-occurrence structures of all data items since the number of combinations of data items that are assumed are enormous. Moreover, the structures can be dynamically changed depending on user behaviors.

### 3.2 Reflection of co-occurrence structures to a data placement by data copy

In the proposed method, by copying data items and storing it in storages other than a storage having an original data item, a data placement reflecting the co-occurrence structures is achieved. If the data item that tends to be required at the same time are placed at the same storage, the probability of getting all required data items together at once, thereby reducing the number of hops required to answer a query.

**Fig 3:**
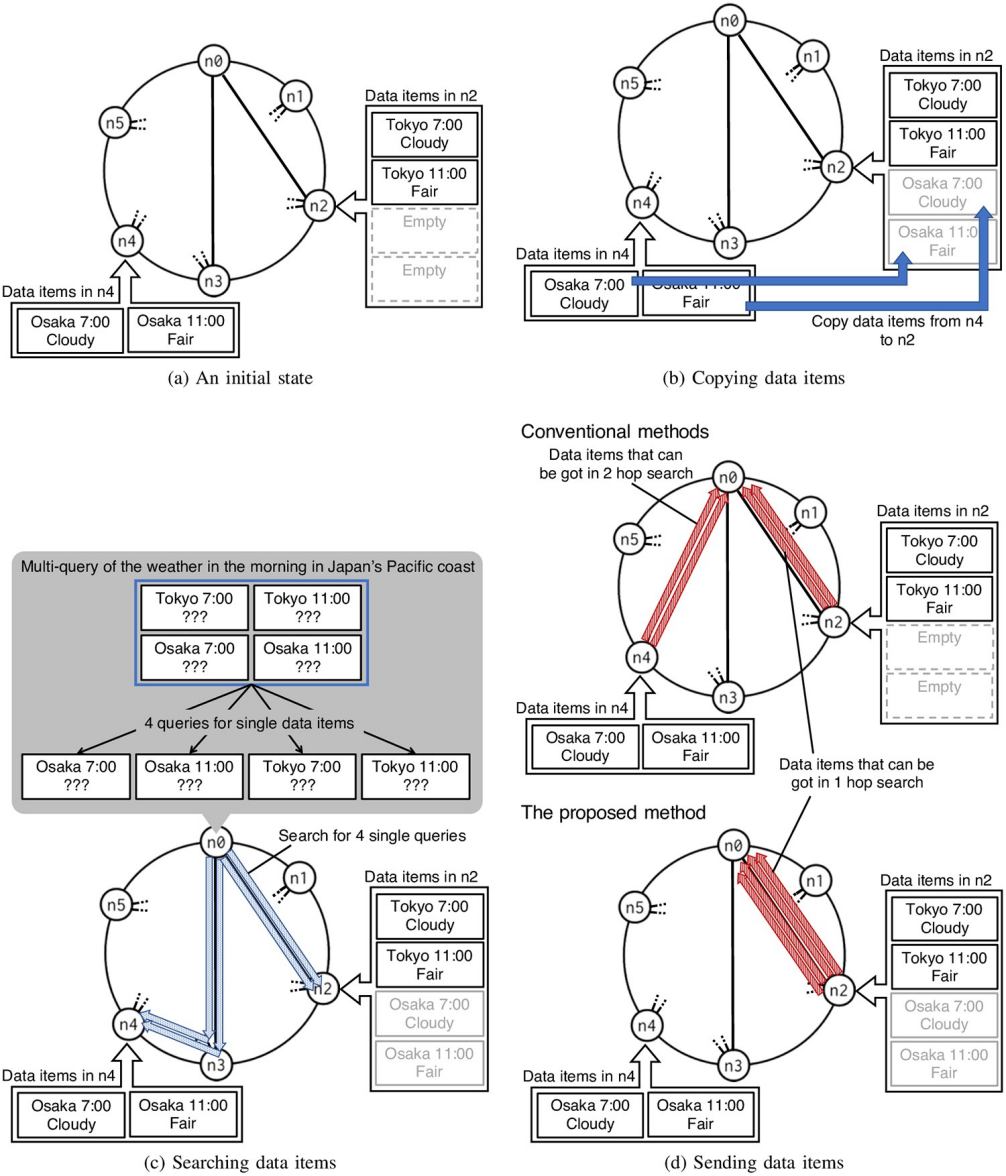
Steps of the proposed method.

The proposed method behaves as an extension of conventional routing methods (including Chord, Chord#, Mercury, etc.). The functions that are added by the proposed method are as follows:

1. From other storages, each storage copies data items that tend to require at the same time with stored data items in the own storage (see Fig 3a and 3b). How to select data items to be copied will be described later.
2. When a multi-query is received, queries for all single data item required by the multi-query are generated (see Fig 3c).
3. When a storage receives queries generated from a multi-query, the storage send the data items to user if the storage has data items required by the multi-query (see Fig 3d).

Most of the conventional methods does not conflict with the proposed method since the original data items are left. If an data item stored in the storages is moved without copy, routing to the data item by the conventional method should be changed in order to maintain reachability to the data item. The proposed method only adds the above functions, and it does not intervene the routing of the conventional method. By using the copy of data items, the co-occurrence structure of the data items can be reflected in the data placement while maintaining the reachability to the data items.

There is a trade-off between the reduction of the number of hops and the storage consumption due to data copy in the proposed method. Needless to say, the proposed method consumes extra storage spaces to store the copies. If the storage space is not limited, all data items on the structured overlay network can be stored at a single storage and queries for every data items can be answered immediately (i.e. 0 hops). Conversely, when the storage space cannot be used, the proposed method corresponds to the conventional method (i.e., the number of hops is not reduced).

**Fig 4:**
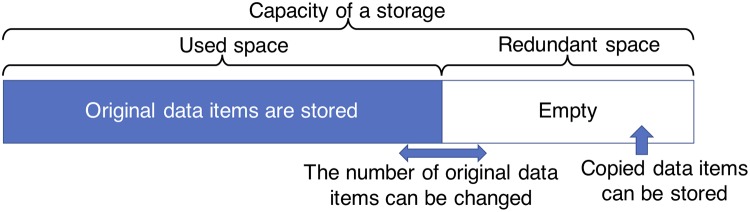
Used and redundant spaces in a storage.

Generally, when data items are stored in a storage, the storage space is not fully utilized, thereby a redundant space exists (see Fig 4). This redundant storage space can be utilized as a space for storing copies of data items in the proposed method. As we mentioned above, copied data items do not affect the reachability of data items, even if it is deleted without any negotiation. When a new data item to be stored appear, we can easily delete the copied data items and store the new data item in the empty space. Therefore, the copied data items do not reduce the potential capacity of the storage. Even if there is no redundant space to store the copied data items, the proposed method never increases the number of hops over that of the conventional method.

In the proposed method, queries received by each storage are recorded as a log, and data items are copied when the number of queries in a log reaches *n*. The data items to be copied are specified by solving an optimization problem (the optimization problem and the algorithm that solves it will be discussed in Section 3.3). As we mentioned in Section 2, structured overlay networks perform in decentralized manner. Not violating the decentralized manner, the data items to be copied are specified by each storage without global information. A storage utilizes only information of a log of queries in its own storage to specify the data items to be copied. A log of queries in a storage is cleared after the copy process, and a storage repeats the copy process every time the number of queries in the log reaches *n*. By updating data items to be copied, we can reflect co-occurrence structures of data items to data placement, even if co-occurrence structures of data items dynamically change.

### 3.3 An optimization problem of data items to be copied

In the proposed method, how to specify the data items to be copied is most important. As we mentioned in Section 3.2, a storage should select the data items to be copied from other storages to its own storage since the storage space is limited. In this section, we will discuss an optimization problem of the data items to be copied when the number of data items that can be copied to the redundant storage space is limited in *c*.

One of naive approaches is to utilize Least Recently Used (LRU) strategy that is commonly used for caching. In this approach, each storage copies latest *c* data items that the storage does not store. Unfortunately, the LRU approach does not achieve good performance (We will show simulation results in Section 4).

**Fig 5:**
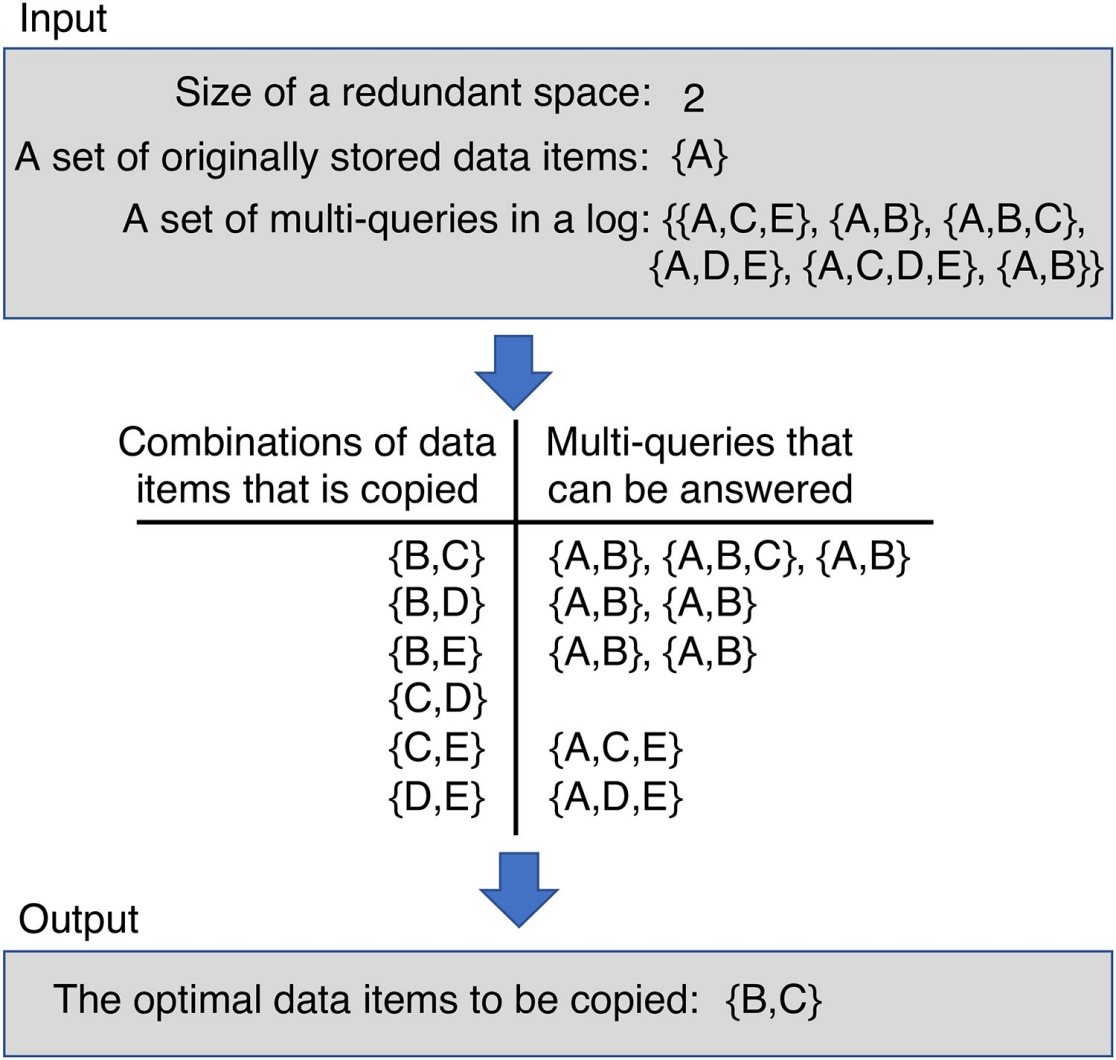
An example of maximizing the number of multi-queries in a log that all queries in the multi-query can be answered by single storage.

In the proposed method, we consider the optimization problem as a problem of maximizing the number of multi-queries in a log that all queries in the multi-query can be answered by single storage. We show an example of the maximization in Fig 5. Suppose that a structured overlay network stores 5 data items with ID A to E, and a storage stores A, originally. When the storage has 2 redundant storage spaces, we have _4_C_2_ choices that are listed in the middle of Fig 5 as the combination of data items to be copied since the number of data items except A is 4. If data items B and C are copied, the storage can answer 3 multi-queries {*A*, *B*} × 2 and {*A*, *B*, *C*} × 1 in a log shown in the top of Fig 5. The number of multi-queries that can be answered when the other combinations of data items are copied, is lower than 3. Therefore, the optimal combination of data items to be copied is *B* and *C*.

This optimization problem can be formulated as the following integer programming.

$$\begin{array}{ccc} \text{maximize} & \sum_{i=1}^{n}x_{i}\\ \text{subject}\;\text{to} & \sum_{j=1}^{m}y_{j}\leq \left|\gamma -\beta \right|+c, & \\ & y_{j}\geq d_{ij}x_{i}, & \forall \;i,j\\ & y_{j}=1, & \forall \;b_{j}\in \gamma . \end{array}$$ (1)

*c*: Size of a redundant storage space.

*α*: A set of multi-queries in a log.

*β*: A set of all data items included in *α*.

*γ*: A set of data items that is originally stored.

*n*: The number of multi-queries in *α*.

*m*: The number of data items in *β*.

*a*_*i*_ ∈ *α*: A set of data items in *i*th multi-query of *α* (1 ≤ *i* ≤ *n*).

*b*_*j*_ ∈ *β*: *j*th data item in *β* (1 ≤ *j* ≤ *m*).

*d*_*ij*_ ∈ {0, 1}: An indicator function that is 1 when *b*_*j*_ ∈ *a*_*i*_, otherwise 0.

*x*_*i*_ ∈ {0, 1}: An indicator function that is 1 when *i*th multi-query in *α* is answered, otherwise 0.

*y*_*j*_ ∈ {0, 1}: An indicator function that is 1 when data item *b*_*j*_ is stored, otherwise 0.

By solving (1), a storage can obtain the optimal combination of data items to be copied. The objective function $\sum_{i=1}^{n}x_{i}$ means the number of multi-queries whose all data items can be answered by the storage. *x*_*i*_ and *y*_*j*_ are explanatory variables. *x*_*i*_ indicates whether *i*th multi-query is answered by the storage or not. *y*_*j*_ indicates whether *j*th data item is stored or not. The relationship between *x*_*i*_ and *y*_*j*_ is described through *d*_*ij*_ in the second inequality constraint. The constraint means that all data items in a multi-query should be stored if the multi-query is answered by the storage. The first inequality constraint represents a space constraint of a redundant storage space.

We propose a greedy algorithm for solving (1), since an integer programming is generally NP-hard. In the greedy algorithm, the efficiency of each multi-query in a log is calculated, and all data items in the multi-query with the maximum efficiency are copied, repeatedly. The pseudo code for the algorithm is shown in Algorithm 1. The input parameters of the algorithm are size *c* of a redundant space, a set *α* of originally stored data items, and a set *γ* of multi-queries in a log. The output of the algorithm is a set *S* of data items to be copied in the redundant space. First of all, data items in a redundant space are cleared (Line 1). Counter *l*_*i*_ counts the number of queries that can be answered by the storage when queries in *a*_*i*_ are copied (Lines 4 to 7). Then, efficiency that is *l*_*i*_ per data item for *a*_*i*_ is calculated (Line 8). A set of data items in *a*_*i*_ that maximizes efficiency $e_{a_{i}}$ is added to a set *S* of data items to be copied in the redundant space (Lines 9 to 10). The above process (Line 3 to 10) is repeated while the redundant space is not fulled.

**Algorithm 1**: A greedy algorithm for (1)

**Input**: *c*, *α*, *γ*

**Output**: The optimal data items to be copied *S*

1 *S* ← ∅

2 **while** |*S*| ≤ *c* **do**

3  **forall** *a*_*i*_ ∈ *α* **do**

4   *l*_*i*_ ← 0

5   **forall** *a*_*j*_ ∈ *α* **do**

6    **if** *a*_*i*_ − *S* − *γ* = *a*_*j*_ − *S* − *γ* **then**

7     *l*_*i*_ ← *l*_*i*_ + 1

8   $e_{a_{i}}\leftarrow l_{i}/\left|a_{i}-S-\gamma \right|$

9  $a_{\text{max}}\leftarrow \text{arg}\;{\text{max}}_{a_{i}\in \alpha }e_{a_{i}}$

10  *S* ← *S* ∪ (*a*_max_ − *γ*)

11 remove recent |*S*| − *c* data items from *S*

12 **return** *S*

## 4 Evaluation

In order to evaluate the performance of the proposed method, we simulated Chord#-based method that is extended by the proposed method. We will confirm the effect of the proposed method on reduction of the number of hops required to answer a query. Additionally, we compare the computation time to solve (1) by using the integer programming and the greedy algorithm.

### 4.1 Simulation settings

In the simulation, we constructed a structured overlay network consisting of 100 storages and generated 10000 multi-queries. The size of the redundant space for storing copied data items is 30 data items per storage. We perform the following 4 protocols:

- **Chord#**: it is original Chord# protocol with a one-dimensional ID space. A load balancing technique [12] is adopted.
- **Chord# with LRU**: Chord# is extended by the proposed method with LRU strategy. Each storage copies data items that are chosen by LRU strategy.
- **Chord# with IP**: Chord# is extended by the proposed method with an exact solution of an Integer Programming (IP). Each storage specifies data items to be copied by directly solving integer programming (1).
- **Chord# with greedy IP**: Chord# is extended by the proposed method with an approximate solution of IP. Each storage specifies data items to be copied by the greedy approach that we mentioned in Section 3.3.

The optimization of data placement is performed every 1000 queries. The number of hops required to answer a query, processing times, and load distribution of storages are compared among the protocols when the system reaches a stationary state. As we mentioned above, the proposed method requires an extra storage space in order to copy data items from the other storages. Hence, it is difficult to fairly compare the proposed method to original Chord#. For fair competition, we compare the proposed method to Chord# with LRU that is a naive approach to utilize a redundant storage space. In the simulations below, the parameters listed in Table 1 will be used as default parameters.

**Table 1:** Default parameters in simulations.

The number of storages	100 storages
Total number of data items	10000 data items
The total number of generated multi-queries	10000 queries
Size *c* of redundant spaces	30 data items
The number *n* of multi-queries in a log	1000 queries

**Fig 6:**
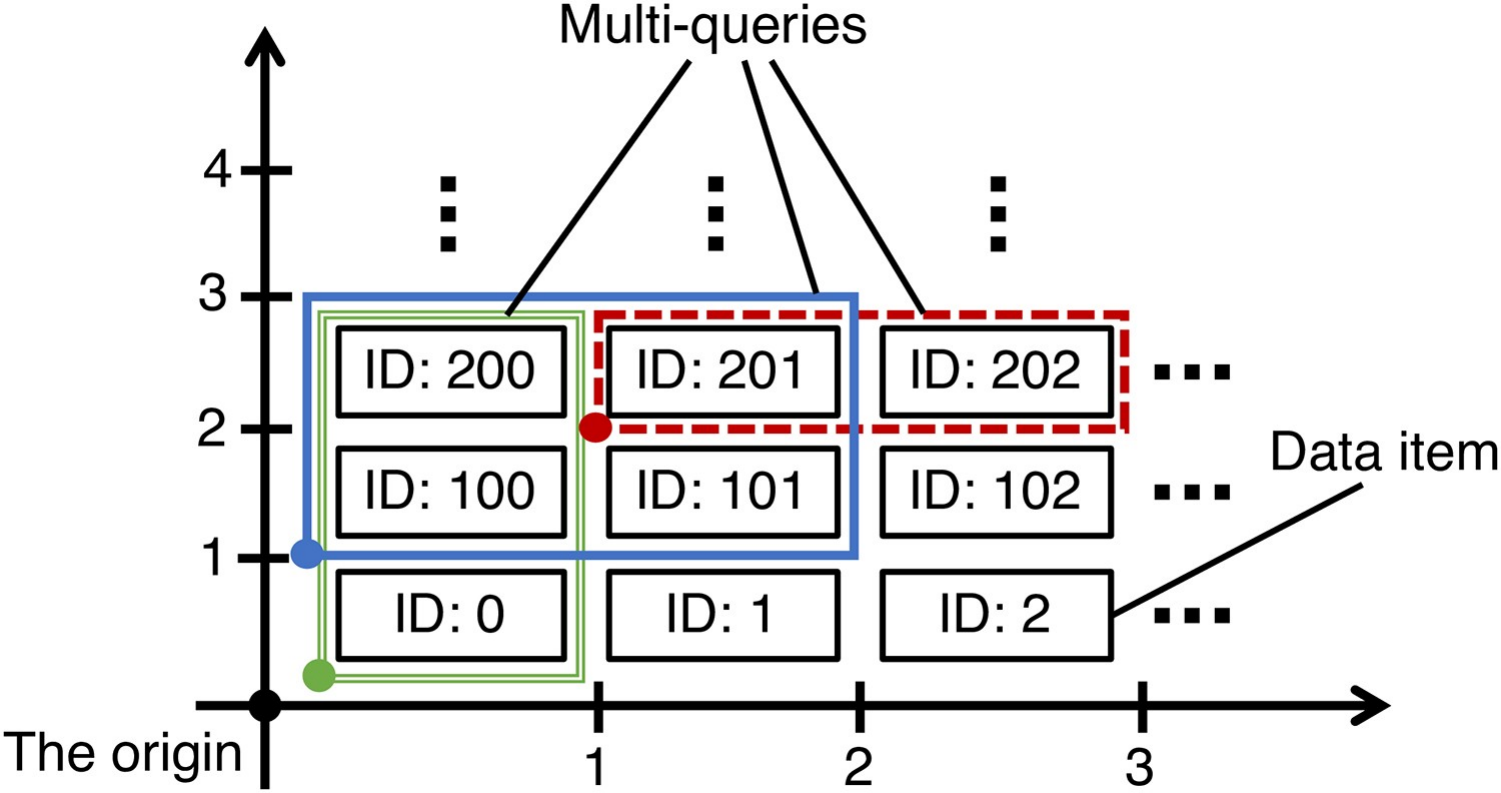
The query generation model expressing co-occurrence structures of data items.

As for the query generation model expressing co-occurrence structures of data items in the simulations, we assume that the co-occurrence structures can be expressed as the two-dimensional torus space shown in Fig 6. We assume that ID on a one-dimensional ID space is assigned to data items stored on the structured overlay network. We map the data items whose IDs are on a one-dimensional ID space, onto the two-dimensional torus space as shown in Fig 6. It is assumed that data items surrounded by an arbitrary rectangle are required as a multi-query. Thereby, data items that are close in the two-dimensional torus space can have high co-occurrence. Though the co-occurrence structure regarding the horizontal direction can be expressed by the ID space, the structure regarding the vertical direction cannot be expressed by the ID space. The rectangle that determines the data items requested as a multi-query is given by 4 parameters: the vertical and horizontal lengths, and the lower left x and y coordinates of the rectangle. The coordinates of the lower left corner of the rectangle are assumed to follow a Zipf distribution with the point of ID 0 as the origin. The vertical and horizontal lengths of the rectangles are also assumed to follow a Zipf distribution, respectively. A shape parameter of each Zipf distribution is set to 1.4. The Power-low and the Pareto principle is frequently observed in network measurements [13]. A Zipf distribution is one of the most common distributions that exhibits the two fundamental characteristics. It is well known that the popularity of files in peer-to-peer file sharing follows a Zipf distribution [14]. By setting the shape parameters to 1.4, the Zipf distributions in our simulation exhibit the Pareto principle: the occurrence probability of the top 20% of the items is almost 80%.

### 4.2 Reduction of the number of hops required to answer a multi-query

**Fig 7:**
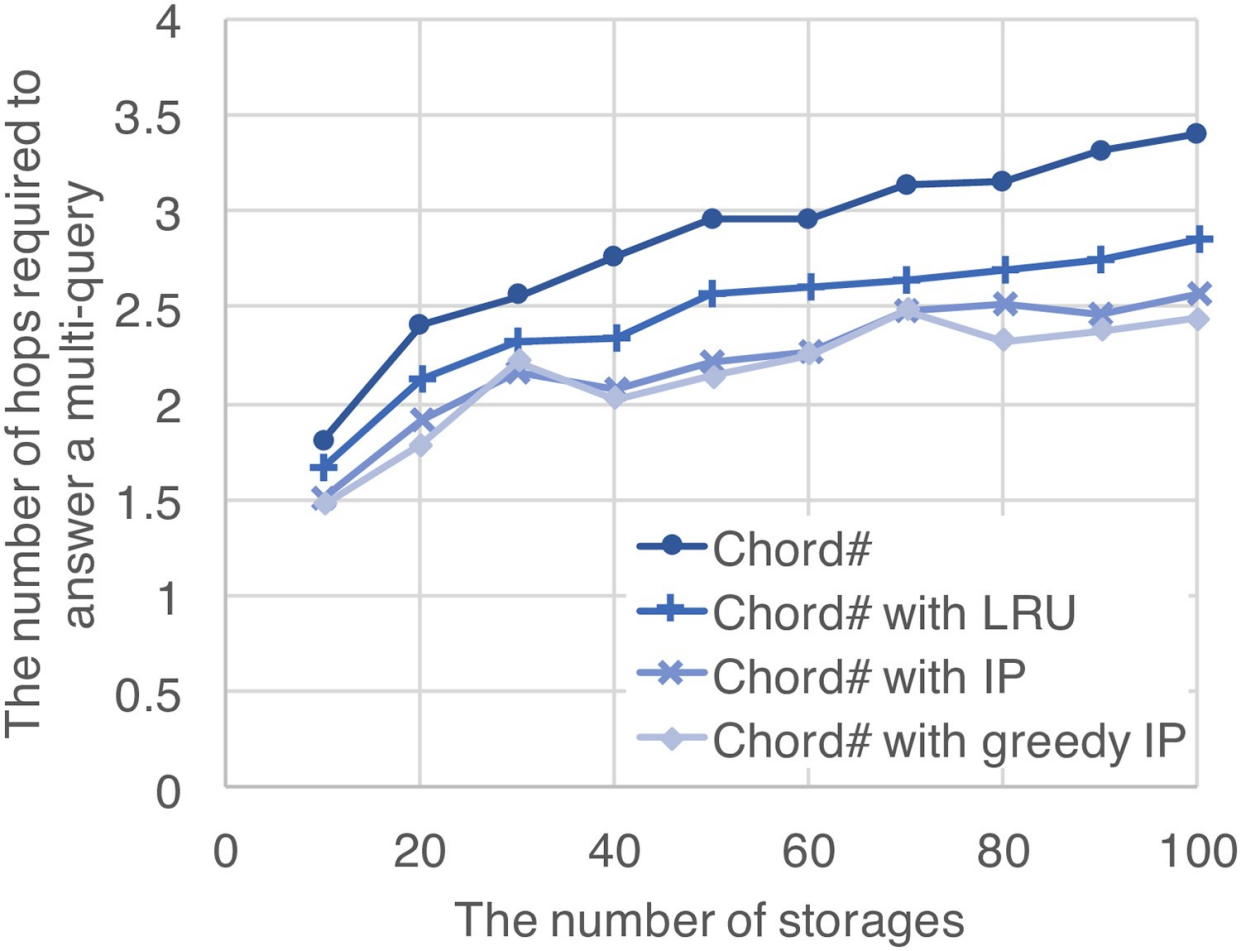
Reduction of the number of hops required to answer a multi-query by the proposed method.

In order to verify reduction of the number of hops required to answer a multi-query by the proposed method, the average number of hops required to answer a multi-query was measured, by changing the number of storages consisting the structured overlay network from 10 to 100 storages. Parameters other than the number of storages are default settings that are shown in Table 1. The average number of hops is defined as the average number of hops required to reach all the data items included in a multi-query. For the 4 protocols, Chord#, Chord# with LRU, Chord# with IP, and Chord# with greedy IP, the results of the average number of hops are shown in Fig 7. The horizontal axis represents the number of storages consisting the structured overlay network, and the vertical axis represents the average number of hops required to answer a multi-query. According to Fig 7, it is confirmed that the average number of hops can be reduced compared with original Chord# in any number of storages when Chord# is extended by the proposed method. In particular, Chord# with greedy IP reduces the average number of hops by about 30% of that of Chord# at the maximum. Moreover, Chord# with IP and Chord# with greedy IP can reduce the average number of hops compared with Chord# with LRU. This is because LRU simply copies the latest data items in a log, and it does not optimize data items to be copied taking the combination of data items in multi-queries into consideration. Apart from that, it is noteworthy that Chord# with greedy IP achieves almost the same performance as Chord# with IP even though the solution of the integer programming problem in Chord# with greedy IP is an approximate solution. A part of the result in Chord# with greedy IP slightly lower than that of Chord# with IP. Since the optimization problem is solved using a log, the results may depend on the future query with randomness even if the solution is optimal. According to the above results, it was confirmed that the average number of hops can be greatly reduced by optimizing data placement with the integer programming rather than a simple approach such as LRU.

Needless to say, the effectiveness of our method depends on the level of co-occurrence. The reduction rate of the average number of hops can be changed depending on a shape parameter of a Zipf distribution in the model of the co-occurrence structure. If there is no co-occurrence structure, the performance of our method will be almost the same as that of the LRU approach.

### 4.3 Effect of redundant storage spaces on load balancing

In the proposed method, since a data item is copied to other storages that do not have the original data item, the storage that answers to the query will be changed, thereby changing the load of the storage. Due to copy of the data item, it is not necessary that the storage with the original data item answers a query. In structured overlay networks, load balancing of storages is important, but load of each storage may be changed due to the proposed method.

**Fig 8:**
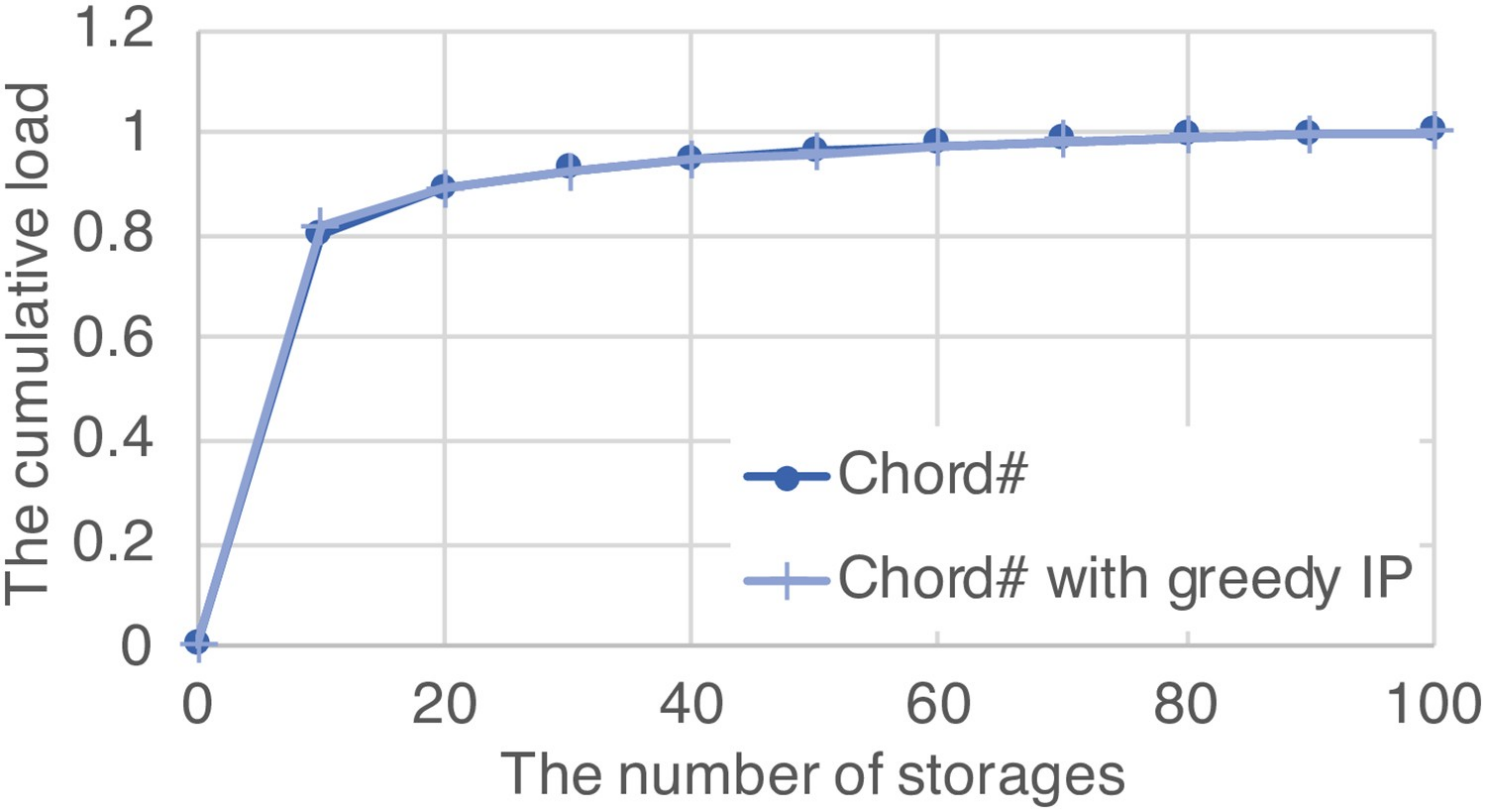
The effect of the proposed method on load balancing.

In order to verify the effect of the proposed method on load balancing of storages, a distribution of loads on storages was derived through a simulation. The default settings that are shown in Table 1 are used in the simulation. The cumulative distribution functions of loads in original Chord# and Chord# with greedy IP are shown in Fig 8. The horizontal axis represents the number of storages, and the vertical axis represents the ratio of the cumulative load of the storages to the total load. Here, the load of each storage is defined as the number of queries that are answered by the storage. Chord# that is the base protocol of the simulation balances the load so that the number of data items stored in each storage is evenly distributed. Note that the number of queries is not evenly distributed though the number of stored data items in each storage is evenly distributed. According to Fig 8, the distribution of load in Chord# with greedy IP is almost the same as that of Chord#, so it can be confirmed that the effect of load change due to the proposed method is negligible.

### 4.4 Reduction of the computation time by the greedy approach

**Fig 9:**
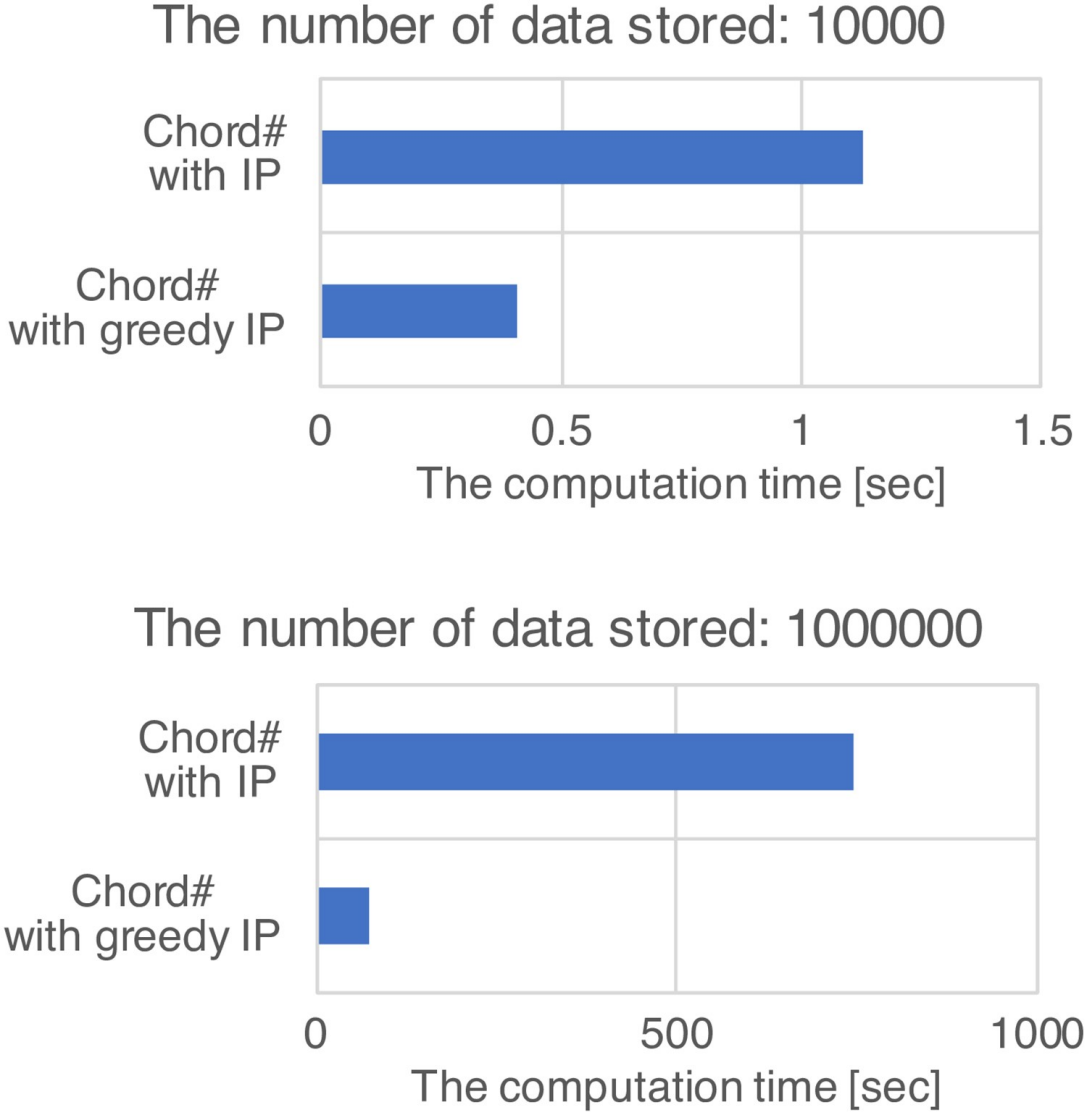
Reduction of the computation time by the greedy approach.

In order to verify the reduction effect of the computation time by the greedy approach, the computation times to solve (1) in Chord# with IP and Chord# with greedy IP were measured when the number of stored data items was 10000 and 1000000. The results are shown in Fig 9. According to Fig 9, we confirmed that the computation time in Chord# with greedy IP is shorter than that in Chord# with IP for both results. Consequently, the greedy approach is about 3 to 10 times faster than the approach in which the exact solution of the integer programming is calculated.

## 5 Related works

A wide variety of protocols have been proposed in researches regarding structured overlay networks. Especially, many studies have focused on the number of hops to search required data items and load balancing across storages [4, 5, 7, 9–12, 15, 16]. Various topologies of overlay networks are tried to reduce the number of hops, and assignment of data items to storages is optimized to balance the load of each storage. In Chord [4] proposed by I. Stoica et al., storages of an overlay network form a ring network. A one-dimensional ID space is assigned to storages on the ring network. Range-queries are not supported though the load is balanced across storages since ID is hashed.

Many protocols have been derived from Chord since it has a simple network structure. Chord# [7] constructs a ring network and a one-dimensional ID space, similar to Chord. Chord# supports range-queries, since values of data items are used as it is, as ID without hashing. The algorithm proposed by D. Karger *et al* is introduced in Chord# as a load balancing technique. Mercury [1] constructs multiple ring networks, called hubs to support multi-attribute queries. In Mercury, near-uniform load balancing is achieved by an algorithm based on a random sampling. Y. Gu *et al* proposed an algorithm that supports range-queries on a distributed tree [17].

For overlay networks other than the structured overlay networks, there are also several researches focusing on the co-occurrence structure of data items. In the literature of semantic overlay networks [18–22], data items are clustered and stored, depending on the contents of the data items. Generally, they are difficult to apply for big data since data items are indexed with metadata.

## 6 Conclusion and future works

We proposed a method to accelerate a response of structured overlay networks by reducing the number of hops required to answer multi-queries. By copying data items from the other storages to a redundant storage space, the proposed method reflects co-occurrence structures of all stored data items onto the data placement in structured overlay networks. We formulated an optimization problem that specifies the data items to be copied as an integer programming, and proposed a greedy approach to solve it. Through simulations, we confirmed that the proposed method can reduce the average number of hops required to answer multi-queries by about 30% at the maximum. Further, the greedy approach in the proposed method can reduce the computation time of the optimization problem by one tenth at the maximum. It is also confirmed that the proposed method does not affect load balancing.

In future work, we will evaluate the proposed method in realistic situations. In order to evaluate scalability of the proposed method, the number of storages should be larger. The query generation model used in the simulations should be replaced by a log of queries in real networks. Moreover, it should be confirmed that various protocols of structured overlay networks except Chord# can be extended by the proposed method without any problems.
